# RNA methylation patterns, immune characteristics, and autophagy-related mechanisms mediated by N6-methyladenosine (m6A) regulatory factors in venous thromboembolism

**DOI:** 10.1186/s12864-024-10294-2

**Published:** 2024-04-24

**Authors:** Deshuai Zhang, Wenxia Fu, Shiwei Zhu, Yitong Pan, Ruogu Li

**Affiliations:** Shanghai Chest Hospital, Shanghai, 200030 China

**Keywords:** Venous thrombosis, Epigenetics, Autophagy, Immune characteristics, Machine learning

## Abstract

Recent studies have found a link between deep vein thrombosis and inflammatory reactions. N6-methyladenosine (m6A), a crucial element in immunological regulation, is believed to contribute to the pathophysiology of venous thromboembolism (VTE). However, how the m6A-modified immune microenvironment is involved in VTE remains unclear. In the present study, we identified a relationship between VTE and the expression of several m6A regulatory elements by analyzing peripheral blood samples from 177 patients with VTE and 88 healthy controls from public GEO databases GSE19151 and GSE48000. We used machine learning to identify essential genes and constructed a diagnostic model for VTE using multivariate logistic regression. Unsupervised cluster analysis revealed a marked difference between m6A modification patterns in terms of immune cell infiltration, inflammatory reactivity, and autophagy. We identified two m6A-related autophagy genes (i.e., CHMP2B and SIRT1) and the crucial m6A regulator YTHDF3 using bioinformatics. We also examined two potential mechanisms through which YTHDF3 may affect VTE. m6A modification, immunity, and autophagy are closely linked in VTE, offering novel mechanistic and therapeutic insights.

**Supplementary Information:**

The online version contains supplementary material available at 10.1186/s12864-024-10294-2.

## Key Points

• The identification of a relationship between VTE and the expression of m6A regulatory elements through analysis of patient samples, highlighting the role of m6A methylation in VTE pathophysiology.

• The development of a diagnostic model using machine learning techniques to predict VTE occurrence, demonstrating the potential for improved early diagnosis and treatment strategies.

• Findings that altered m6A methylation patterns influence immune cell infiltration, inflammatory reactivity, and autophagy in VTE, suggesting new therapeutic targets.

• The identification of key m6A-related autophagy genes and their mechanisms, providing insights into the complex interactions between RNA methylation, immune response, and autophagy in VTE.

• Implications of the study for the development of innovative therapeutics for VTE, emphasizing the potential of targeting m6A regulatory factors and their mediated mechanisms.

**Supplementary Information:**

The online version contains supplementary material available at 10.1186/s12864-024-10294-2.

## Introduction

Venous thromboembolism (VTE), which comprises deep vein thrombosis (DVT) and pulmonary embolism (PE) [1], is the third most prevalent cardiovascular disease (CVD) worldwide after hypertension and coronary heart disease [2]. Each year, approximately 600,000 cases are diagnosed with VTE in the United States [3]. VTE can also lead to several complications, including recurrence, persistent thromboembolic pulmonary hypertension, postthrombotic syndrome, and mortality [4–8]. Therefore, early diagnosis and treatment of VTE is crucial to significantly minimize mortality and improve prognosis.

Since its discovery in 1974, m6A modification has been the most widespread epigenetic modification of RNA in eukaryotic cells [9, 10]. Without changing the base sequence, this dynamic and reversible methylation process can affect RNA transcription, splicing, degradation, and translation [11], thereby playing a role in the onset of numerous diseases. Methyltransferases, including METTL3, METTL14, and WTAP, catalyze the methylation of m6A [12], whereas demethylases, such as FTO protein and ALKBH5, demethylate the changed bases [13]. Furthermore, m6A methylated reader proteins are RNA-binding proteins that can specifically adhere to m6A methylation sites [14] and modify RNA secondary structure to influence protein‒RNA interactions [15].

The development of VTE is closely related to the immune system, as evidenced by the literature indicating the involvement of immune cells, such as neutrophils and monocytes, in all stages of the disease [16]. Inflammatory reactions also greatly facilitate the progression of VTE [17]. The connection between m6A and the immune system has been well established, with extensive research [10, 18–20] confirming the regulatory effect of m6A on various autoimmune diseases. Immune cells, such as neutrophils [21] and monocytes [22], exhibit unique m6A modification patterns that can affect their functions. In addition, m6A modification has been reported to affect inflammatory reactions [23]. Analyzing changes in the m6A modification pattern of patients with VTE is crucial. The impact of these changes on the immune microenvironment relevant to VTE must be examined to elucidate the pathogenesis of VTE and explore new directions for immune molecular therapy research.

We hypothesized that m6A alteration modulates the immunological microenvironment, thereby influencing the incidence and development of VTE. We aimed to investigate the effect of m6A methylation on the immune microenvironment and its potential molecular mechanisms in VTE using bioinformatic analyses of public databases and our own sequencing data.

## Materials and methods

### Collection and preprocessing of raw data

**Fig. 1:**
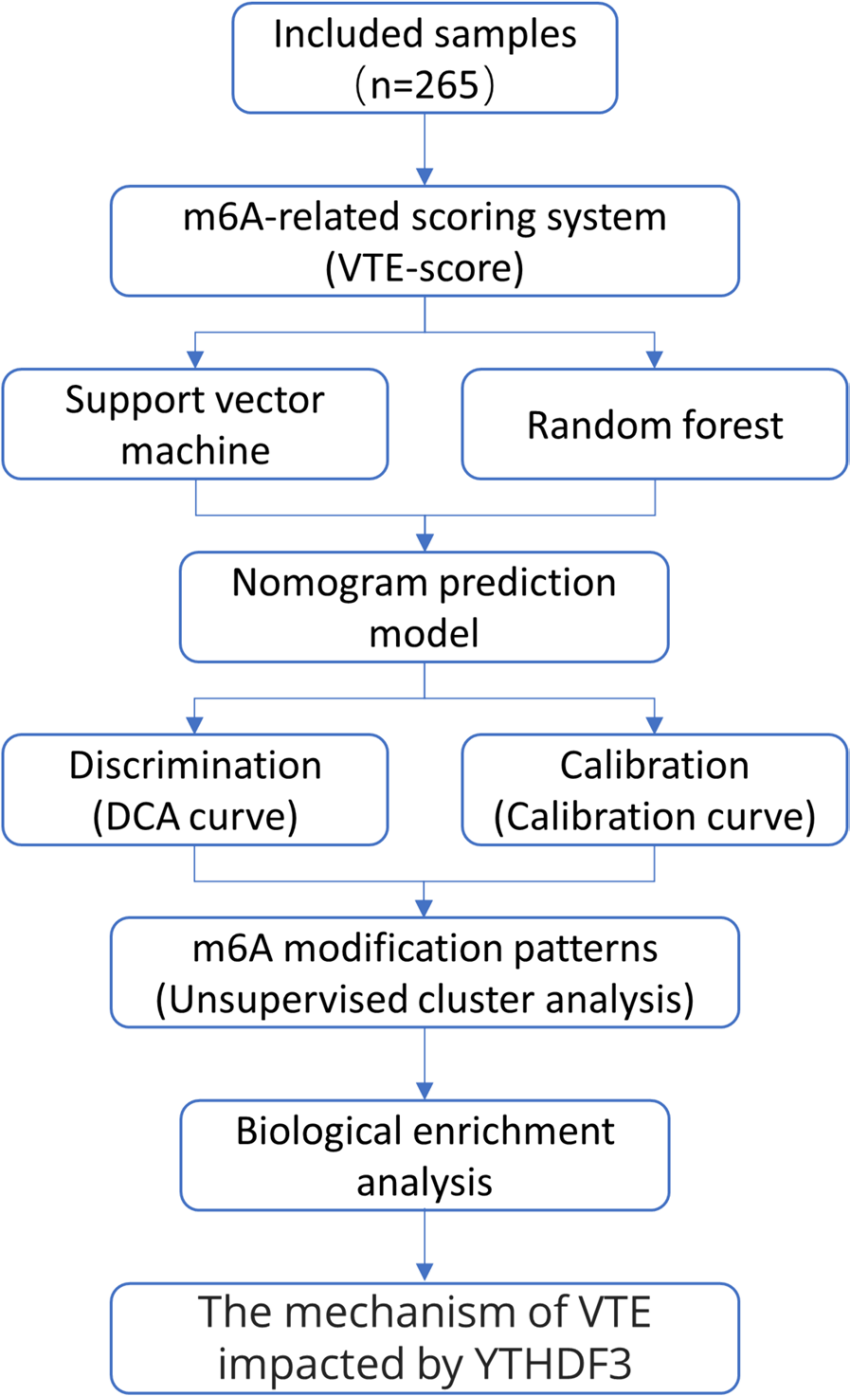
Figure 1 presents a flowchart of the study, outlining the progression from sample inclusion to the analysis of YTHDF3’s impact on VTE via m6A methylation patterns and machine learning models

Figure 1 illustrates the procedure of this study. The GSE19151 profile of human expression, which includes the entire peripheral blood expression array of 70 patients with VTE and 63 healthy controls, was derived from the Gene Expression Omnibus (GEO) public database [24]. GSE19151 was derived from GPL571 (affymetrix human genome u133a 2.0 array). Furthermore, GSE48000 was retrieved, which contained 132 whole blood samples, including 107 VTE and 25 control samples. GSE48000 was derived from GPL10558 (Illumina human ht-12v4.0 expression beadchip). Clinically relevant information for GSE48000 was collected. All probes were labeled with the gene names to which they corresponded; probes without labels were removed. Quantile normalization was then applied to the expression data using the limma program [25]. The proxy V-variable analysis (sva) program was subsequently used to remove batch differences. The related website address is listed in Additional File 1.

### Identification of VTE-associated m6A regulators

Using the limma package and cutoff criteria of adjusted p*-*value of 0.05 and |log2(fold change)| > 1 [25], we identified m6A regulators with expression differences between the VTE and control groups [25]. Pearson correlation analysis was then performed to evaluate and quantify the force of the association between the previously discovered m6A regulatory variables [26–28] (Additional file 2). The listed genes were used in the subsequent investigation.

### Diagnostic model

We employed random forest (RF) and support vector machine (SVM) models to determine the optimal mathematical classification scheme for diagnosing VTE. The SVM model estimated the significance of variables using w2, the value of the discriminant function coefficient, whereas the RF model used a lower Gini coefficient. These two models were implemented using the randomForest and kernlab tools. The residual error values and areas under the receiver operating characteristic curve (AUCs) of the two models were compared using the pROC and DALEX software to determine which model was superior. After performing dimension reduction and feature selection, we added the selected m6A regulatory components to a prediction model using logistic regression analysis. We then calibrated the consistency between the model’s predicted and actual values using a calibration chart and assessed the diagnostic effectiveness of the prediction model using the ROC curve [29].

### Relationship between immunological properties and m6A regulators

The prevalence of 23 infiltrating immune cells in different groups was assessed using the GSVA package [30, 31] and single-sample gene set enrichment analysis (ssGSEA) [32] (Additional file 3). The enrichment scores that describe the relative abundance of every immune cell in the VTE and control groups were compared using a t-test. In addition, the immune response gene set from the ImmPort database [33] (Additional file 4) and the list of genes associated with inflammation from the HGNC database [34] (Additional file 5) were used to evaluate immune system activity.

### Unsupervised cluster analysis of m6A alteration patterns in VTE

We used unsupervised pattern clustering to separate VTE samples into different m6A modification patterns according to the expression of 16 m6A regulatory variables. The ideal cluster number [35, 36] was determined using the ConsensusClusterPlus program to generate a k value of 2–9 and the delta area fraction-corresponding cumulative distribution function curve. To test the clustering impact of the two m6A-modified subgroups, principal component analysis (PCA) was performed. Furthermore, we identified genes regulated by m6A regulatory factors by comparing the differentially expressed genes (DEGs) of VTE samples from various m6A clusters (*p* < 0.05). Relevant DEGs were used for further investigation.

### Analysis of biological enrichment for distinct m6A clusters

We performed functional enrichment analysis, including GO enrichment analysis and Kyoto Encyclopedia of Genes and Genomes (KEGG) pathway analysis, to further examine the biological function of DEGs. The org.Hs.eg.dbR package was used to mark genes for GO enrichment analysis, and clusterProfiler was used for enrichment analysis with a *p* < 0.05 criterion. KEGGrestAPI was used to obtain gene annotations for the KEGG pathway analysis, and with a statistical threshold of 0.05, enrichment analysis was performed using clusterProfiler.

### Identification of genes mediated by m6A

After clustering, we identified “genes regulated by m6A regulatory factors” as DEGs in two independent sets of VTE samples with m6A modification. We used weighted gene coexpression network analysis (WGCNA) to identify important genes by distinguishing coexpressed gene modules and examining the relationship between gene networks and relevant symptoms. To analyze the gene expression profile of the VTE sample (*n* = 177) using the WGCNA package, we used the goodSamplesGenes function to eliminate gene and sample outliers. The correlation between the different modules and subgroups was determined using Pearson correlation analysis. By further assessing the connection between the m6A modification pattern and gene expression, we identified gene significance (GS) and module membership (MM). The threshold for the hub gene was previously established to be |MM|>0.8 and |GS|>0.1 [37].

### Statistical analysis

Statistical analyses were performed using R (v4.2.2) and Bioconductor (Additional file 1). For each collected data point, a two-sided statistical test procedure was applied, and 0.05 was always chosen as the statistical threshold. *P < 0.05, **P < 0.01, ***P < 0.001 indicated a statistically significant difference.

## Results

### Variability in m6A regulator genes and immune activation of VTE

**Fig. 2:**
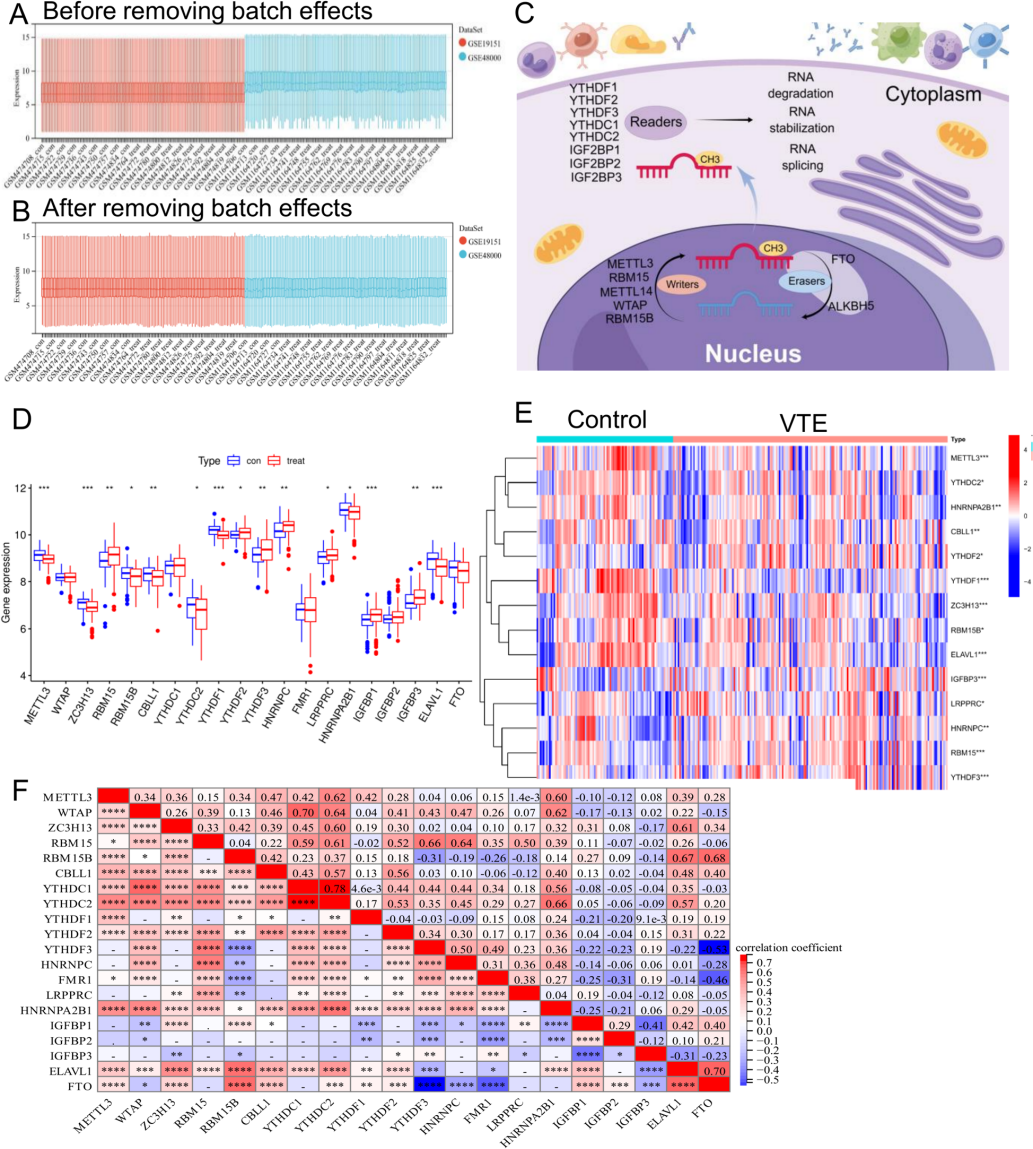
Panels **A**, **B** show the consistency of gene expression data before and after correcting for batch effects. Panel **C** illustrates the m6A RNA methylation process in the cell. Panel **D** displays differential expression of m6A regulators between control and VTE. Panel E compares gene expression patterns between control and VTE subjects. Panel F shows the correlation between different m6A regulators

The inSilicoMerging and sva packages were used to merge data and reduce batch effects, respectively. After processing, the data distribution across the datasets was consistent (Fig. 2A–B), indicating that the effect of batch processing was eliminated. We evaluated 26 m6A regulatory variables and created a graphic to show the procedure of m6A alteration in the immunological microenvironment (Fig. 2C, drawn by Figdraw, and Additional file 2). The expression profiles of m6A regulatory factors were then isolated from the training set, and 20 m6A regulators were identified (Fig. 2D). HNRNPA2B1 exhibited a greater baseline expression level than other m6A regulatory factors. The expression of 15 regulators, including METTL3, ZC3H13, RBM15, RBM15B, CBLL1, YTHDC2, YTHDF1, YTHDF2, YTHDF3, HNRNPC, LRPPRC, HNRNPA2B1, IGFBP3, IGFBP1, and ELAVL1, varied considerably between the VTE and control groups. ELAVL1 experienced the greatest absolute change, followed by RBM15B (*P* < 0.001). In contrast, the gene expression levels of METTL3, ZC3H13, RBM15B, CBLL1, YTHDC2, HNRNPA2B1, and ELAVL1 were dramatically reduced in VTE. The heatmap presented in Fig. 2E shows the 14 DEG patterns. Then, to establish the relationship between various m6A modulators, we conducted a correlation study. Positive correlations were observed between METTL3 and YTHDC1, YTHDC2, and HNRNPA2B1 (Fig. 2F), which may be associated with the recruitment of these proteins by METTL3 [38, 39]. The strongest correlation coefficient (*r* = 0.79) was found between YTHDC1 and YTHDC2.

### Construction of a patient-specific scoring system for m6A methylation modification patterns

**Fig. 3:**
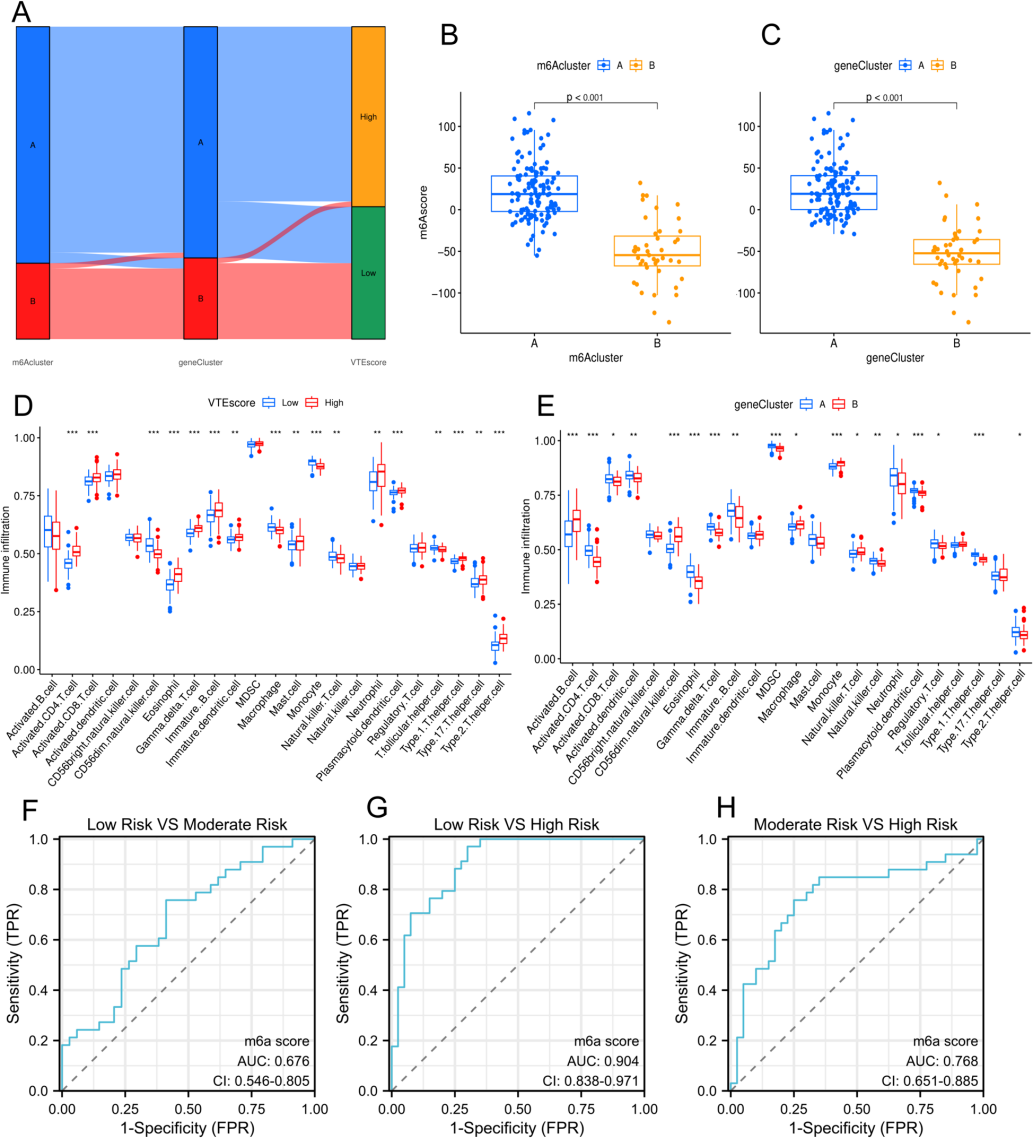
Figure 3 illustrates the m6A methylation pattern analysis in VTE. Panel **A** shows the distribution of m6A and gene clusters in relation to VTE scores. Panels **B** and **C** depict the VTE score differences between m6A clusters A and B, while panels **D** and **E** correlate VTE scores with immune infiltration. Panels F, G, and H show ROC curves evaluating the VTE risk differentiation based on low, moderate, and high VTE scores, respectively

The methylation pattern of m6A was carefully measured in each patient, and 14 genes were identified as being linked to prognosis based on univariate Cox analysis of 26 genes of the m6A methylation pattern (Fig. 2E). A patient-specific scoring system for DVT called the VTE score, was constructed using 14 prognostic genes of the m6A methylation pattern and PCA to evaluate each patient’s m6A methylation modification pattern. The scoring system successfully evaluates the methylation level of m6A in patients, considering the heterogeneity of individual patients. According to the median, patients were separated into two groups: those with a high VTE score and those with a low VTE score. The Sankey diagram demonstrates the variations in the m6A cluster, gene cluster, and VTE score of individual patients, demonstrating the consistency and dependability of our analytical results (Fig. 3A).

According to Fig. 3B, the high VTE score of gene cluster A was likely associated with immunity because gene cluster B has a significantly lower VTE score than gene cluster (A) Additionally, as shown in Fig. 3C, m 6A cluster A had a higher VTE score than m6A cluster (B) Further studies have revealed a correlation between an elevated VTE score and immune infiltration (Fig. 3D and E). These results indicate that a high VTE score is associated with the immune response and can be used to assess the methylation modification pattern of m6A in individuals to estimate the risk of DVT.

Next, we attempted to use the VTE score to differentiate patient risks. We evaluated the VTE risk based on the clinical data of 107 patients from the GSE48000 dataset using the following criteria: [1] “low-risk” individuals experienced one provoked VTE; [2] “moderate-risk” patients experienced no more than one unprovoked VTE, and [3] “high-risk” patients experienced two unprovoked VTEs. According to these criteria, 107 patients were classified as follows: 34 as low-risk, 33 as medium-risk, and 40 as high-risk. With AUCs of 0.68, 0.90, and 0.77, respectively, we separated low-risk individuals from high-risk individuals, low-risk individuals from medium-risk individuals, and medium-risk individuals from high-risk individuals using VTE score prediction analysis (Fig. 3F–H). These results demonstrated that the VTE score prediction model we developed has excellent stability and reliability in identifying the risk of DVT and embolism among patients.

### Development and assessment of a diagnostic model based on m6A

**Fig. 4:**
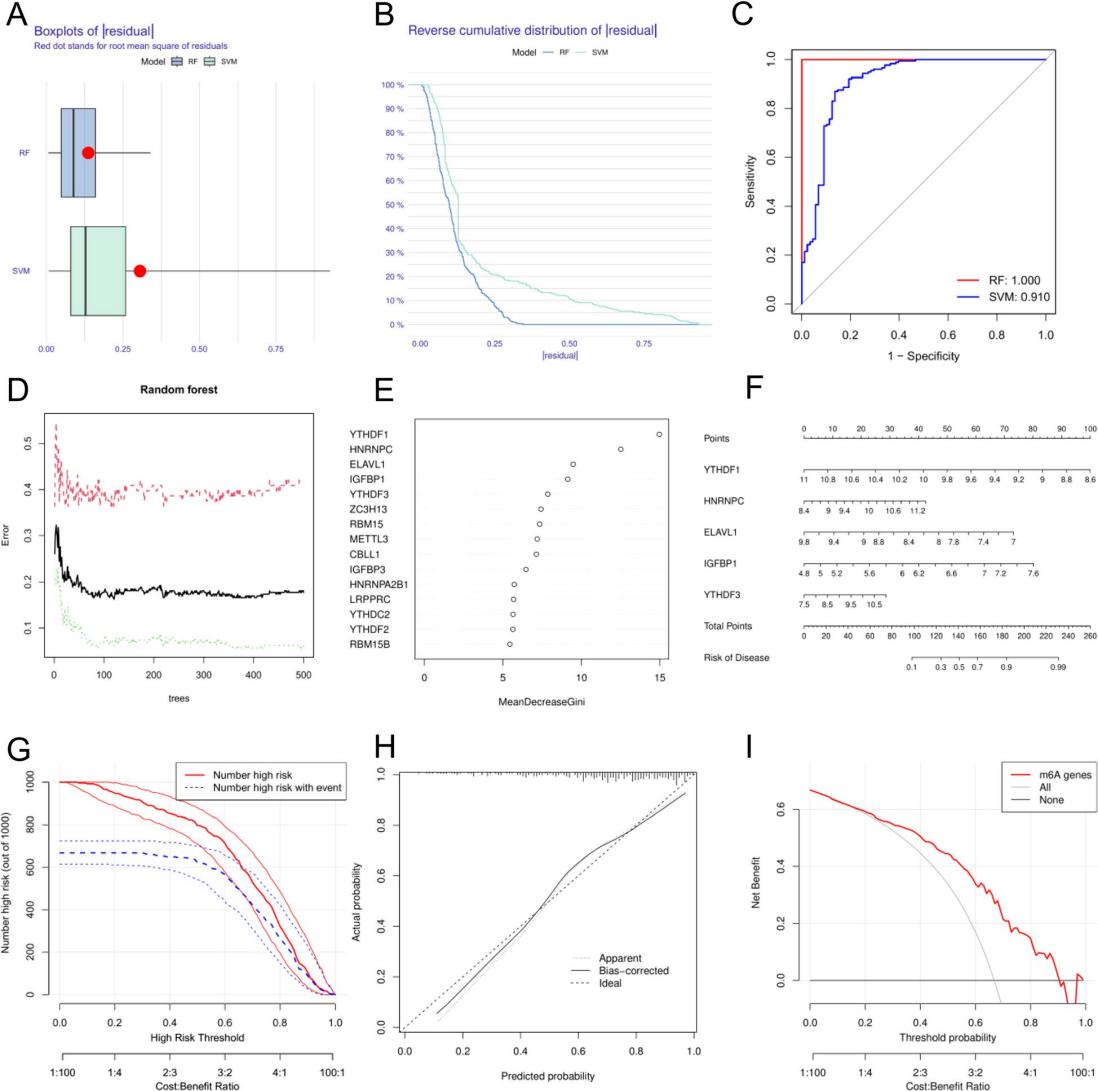
Model accuracy comparison using residuals for random forest (RF) and support vector machine (SVM) (**A**, **B**); receiver operating characteristic (ROC) curve indicating superior performance of RF (**C**); error rate reduction over RF iterations (**D**); importance of m6A regulatory variables (**E**); nomogram for VTE risk prediction (**F**); and model validation through discrimination and calibration curves (**G**-**I**).

To further narrow the scope of m6A regulatory factors, we used the RF and SVM methods to construct two models using 14 differentially expressed m6A regulatory factors. The median residual derived by the RF algorithm was lower than that of the SVM, indicating that the RF model was more accurate (Fig. 4A and B). As shown in Fig. 4C, the AUC value of the random forest model was also greater than that of the SVM model. The number of RF iterations was plotted against the classification error, and it was found that as the number of iterations exceeded 300, the classification error became modest and steady (Fig. 4D). We ranked the significance of 15 m6A regulatory variables using the random forest model (Fig. 4E). We then used RMS to create a nomogram model based on the five most significant regulatory elements chosen as predictors. The resulting design is illustrated in Fig. 4F. We evaluated the discrimination and calibration abilities of the nomogram using DCA, calibration, and clinical impact curves (Fig. 4G–I). The calibration curve revealed that the difference between the observed and predicted values was modest, indicating that the nomogram model had a high predictive value. These findings suggest that YTHDF1, HNRNPC, ELAVL1, IGFBP1, and YTHDF3 play a significant role in the occurrence of VTE.

### Immune microenvironment and the connection between m6A regulators

**Fig. 5:**
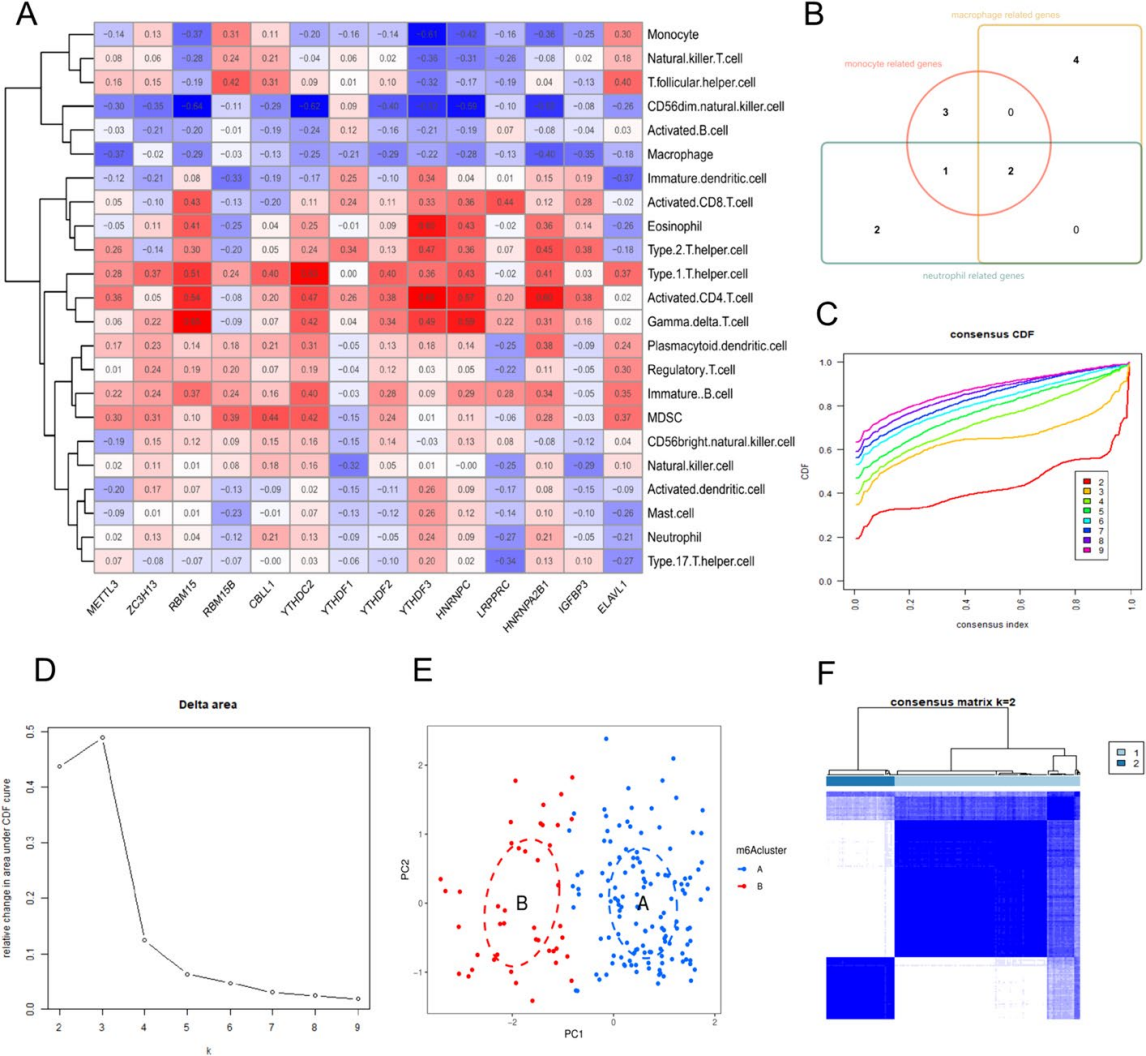
This Figure presents the analysis of m6A modification patterns in VTE: (**A**) Correlation matrix of m6A regulatory factors with immune cell types. **B** Venn diagram indicating the relationship between inflammatory response-related genes and m6A regulatory factors. **C** Consensus clustering cumulative distribution function (CDF) for determining the number of clusters. **D** Delta area plot to assist in selecting the optimal number of clusters. **E** PCA plot illustrating two distinct m6A modification patterns. **F** Consensus matrix heatmap confirming the clustering of samples into two distinct groups

Based on recent studies [40, 41] demonstrating the regulatory use of m6A modification in the immune microenvironment and immune response, we performed a correlation analysis (|R| > 0.2, *p* < 0.05) to investigate the role of m6A regulatory factors in the immune microenvironment. We found a positive correlation between monocyte abundance and REM15B and ELAVL1 and a negative correlation between monocyte abundance and REM15, YTHDF3, HNRNPC, and HNRNPA2B1. A correlation between macrophages and six m6A regulatory factors (i.e., METTL3, RBM15, YTHDC2, YTHDF3, HNRNPA2B1, and IGFBP3) was also observed. Neutrophils, the most prevalent immune cells involved in acute inflammatory responses, were found to be positively associated with CBLL1, HNRNPA2B1, and YTHDF3 but negatively associated with LRPPRC and ELAVL1 (Fig. 5A). Using a Venn diagram to illustrate the relationship between the predicted inflammatory response-related genes and the changed m6A regulatory factors (Fig. 5B, Additional File 6), we identified two m6A regulatory factors (i.e., YTHDF3 and HNRNPA2B1) that may play a crucial role in the inflammatory response. HNRNPA2B1 is a member of the nuclear RNA-binding protein (RBP) family, referred to as heterogeneous nuclear ribonucleoproteins (hnRNPs) [42].

### Expression types determined using 14 m6A methylation modification factors

**Fig. 6:**
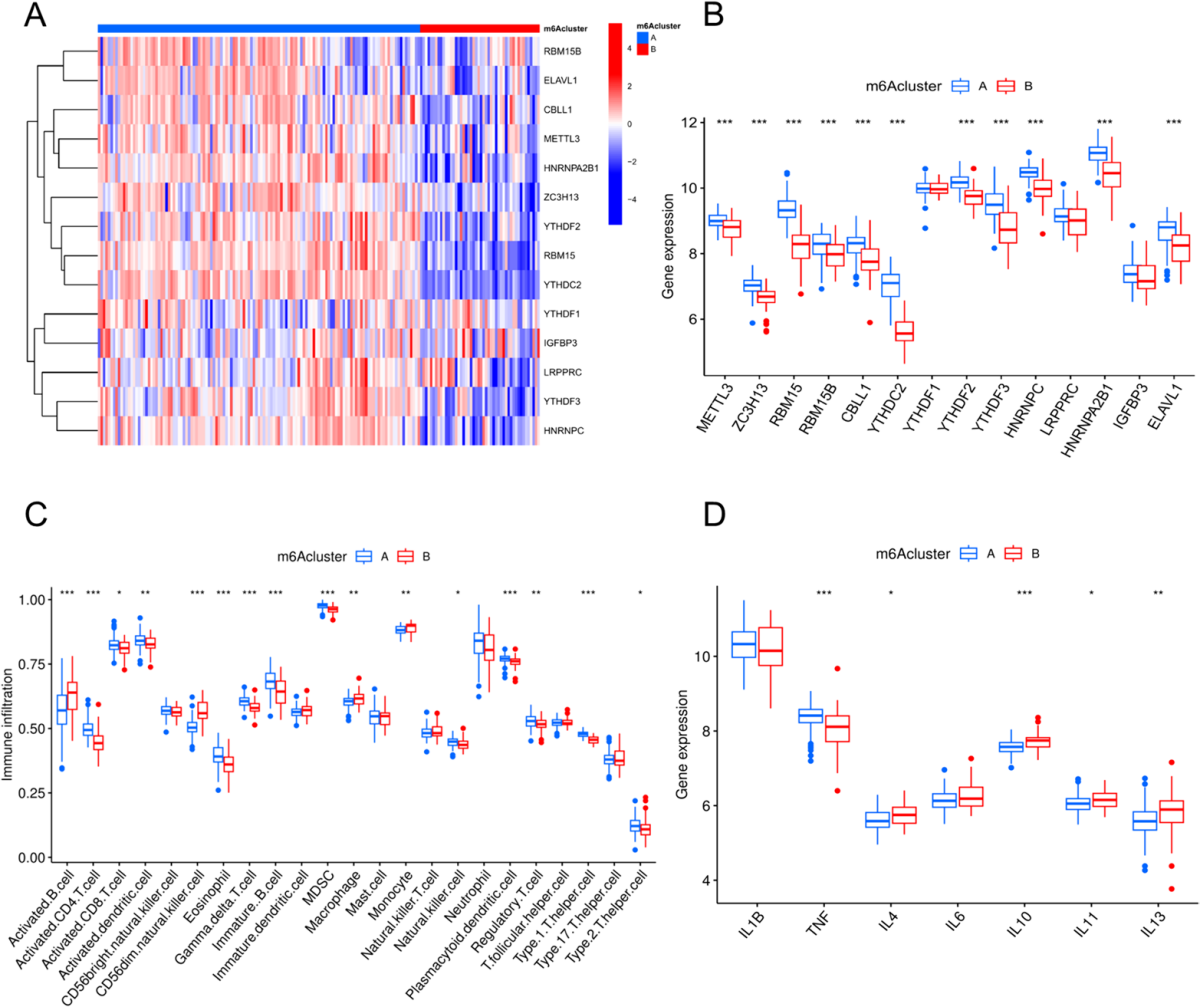
It displays the differential analysis of m6A regulators in VTE: (**A**) Heatmap showing expression levels of m6A regulators across samples. **B** Boxplots indicating significant differences in expression of m6A regulators between two m6A modification patterns. **C** Boxplots showing the variation of immune infiltration between the two m6A modification patterns. **D** Expression levels of selected inflammatory markers across the m6A modification patterns

We used unsupervised clustering to categorize datasets into different groups and discovered various m6A modification patterns in VTE based on the expression profiles of 14 m6A regulators (Fig. 5C–E). We identified two unique patterns, with subgroup A containing 134 samples and subgroup B containing 43 samples, assuming that k = 2 is the ideal value. We visually represented the similarity between data from multiple groups using PCA, which showed two unique modification groups for m6A (Fig. 5F). Eleven m6A regulators exhibited altered expression levels between the two subgroups, as determined by differential analysis of the two patterns (Fig. 6A and B). Subgroup A exhibited higher levels of METTL3, ZC3H13, RBM15, RBM15B, CBLL1, YTHDF1, YTHDC2, YTHDF3, HNRNPC, HNRNPA2B1, and ELAVL1 than subgroup B. These findings suggest a similarity in the pattern of m6A alteration in VTE, which might serve as a reference for future studies.

To examine the relationship between the m6A modification type and the immune environment, we evaluated the degree of immune cell infiltration and identified two distinct immune cell composition models A and B (Fig. 6C). Mode A had elevated amounts of activated CD4 + T cells, activated CD8 + cells, eosinophils, immature B cells, neutrophils, dendritic cells, and numerous T helper cells, whereas Type B had higher levels of infiltrated-activated B cells and CD56 + natural killer cells and marginally greater levels of the mononuclear macrophage system. This indicates that different immune cells have distinct m6A alterations. Figure 5E shows our analysis of the variable expression of inflammatory response-related genes in two m6A modification modes using information from the HGNC database [34]. A proinflammatory factor (TNF-β) and four anti-inflammatory factors (i.e., IL-4, IL-10, IL-11, and IL-13) were expressed at greater levels in mode A than in mode B (Fig. 6D). The mode B immunophenotype, which indicates a heightened intravascular inflammatory response, may result in the formation of more vascular thrombosis, leading to a poorer prognosis.

### Biological properties of distinguishable m6A modification types

**Fig. 7:**
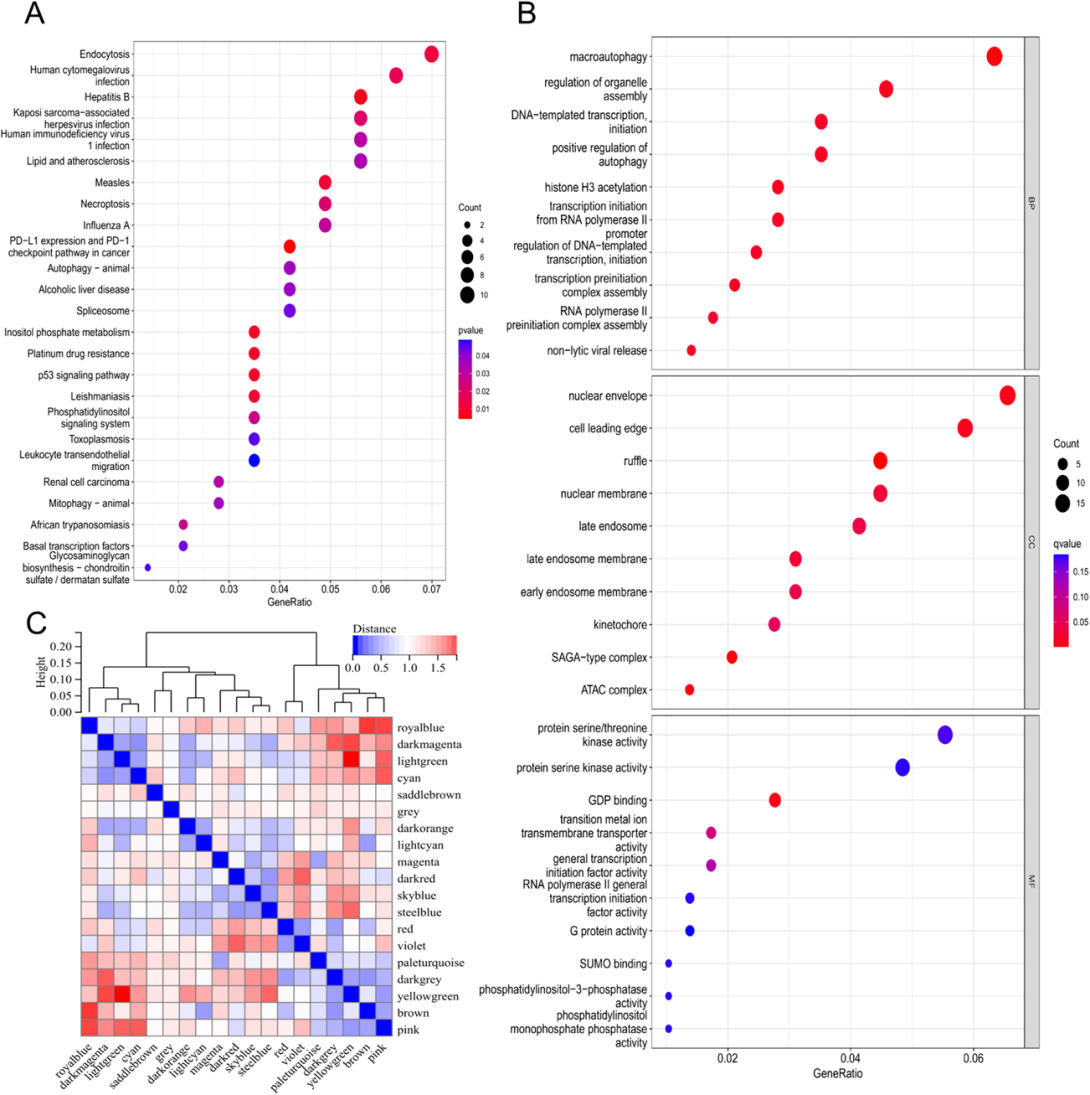
Figure 7 displays the GO enrichment analysis of differentially expressed genes between two m6A-modified subgroups. Panel **A** shows a bubble chart with enrichment in biological processes like endocytosis and immune response. Panel **B** highlights the autophagy signaling pathway as a dominant mechanism in a bubble chart. Panel **C** presents a heatmap of enriched terms across m6A modification patterns, indicating associations with various cellular and molecular functions

We performed a functional enrichment analysis to investigate the significance of the m6A alteration pattern in VTE in more detail. We identified 307 genes with differential expression between the two m6A-modified subgroups. The GO enrichment analysis revealed that these DEGs were mostly associated with endocytosis, immunological response, lipid metabolism, and atherosclerosis (Fig. 7A and Additional file 7).

The findings of the present study show a relationship among m6A methylation alteration factors and the immune microenvironment and autophagy in VTE. The differences between the two m6A subgroups revealed that the autophagy signaling route was the dominant mechanism (Fig. 7B). Autophagy is the degradation of protein macromolecules and organelles by autophagosomes in eukaryotic cells. Under physiological conditions, autophagy is essential for cell remodeling and intracellular balance maintenance. However, under pathological conditions, such as inflammatory responses and oxidative stress, autophagy is overactivated in the process of cell death; hence, controlling intracellular autophagy activity is of utmost importance [43]. Furthermore, most cells of cardiovascular origin, including cardiomyocytes, endothelial cells, and arterial smooth muscle cells [44], rely on autophagy to maintain intracellular homeostasis, and autophagy is strongly associated with the pathophysiology of cardiovascular diseases [45].

**Fig. 8:**
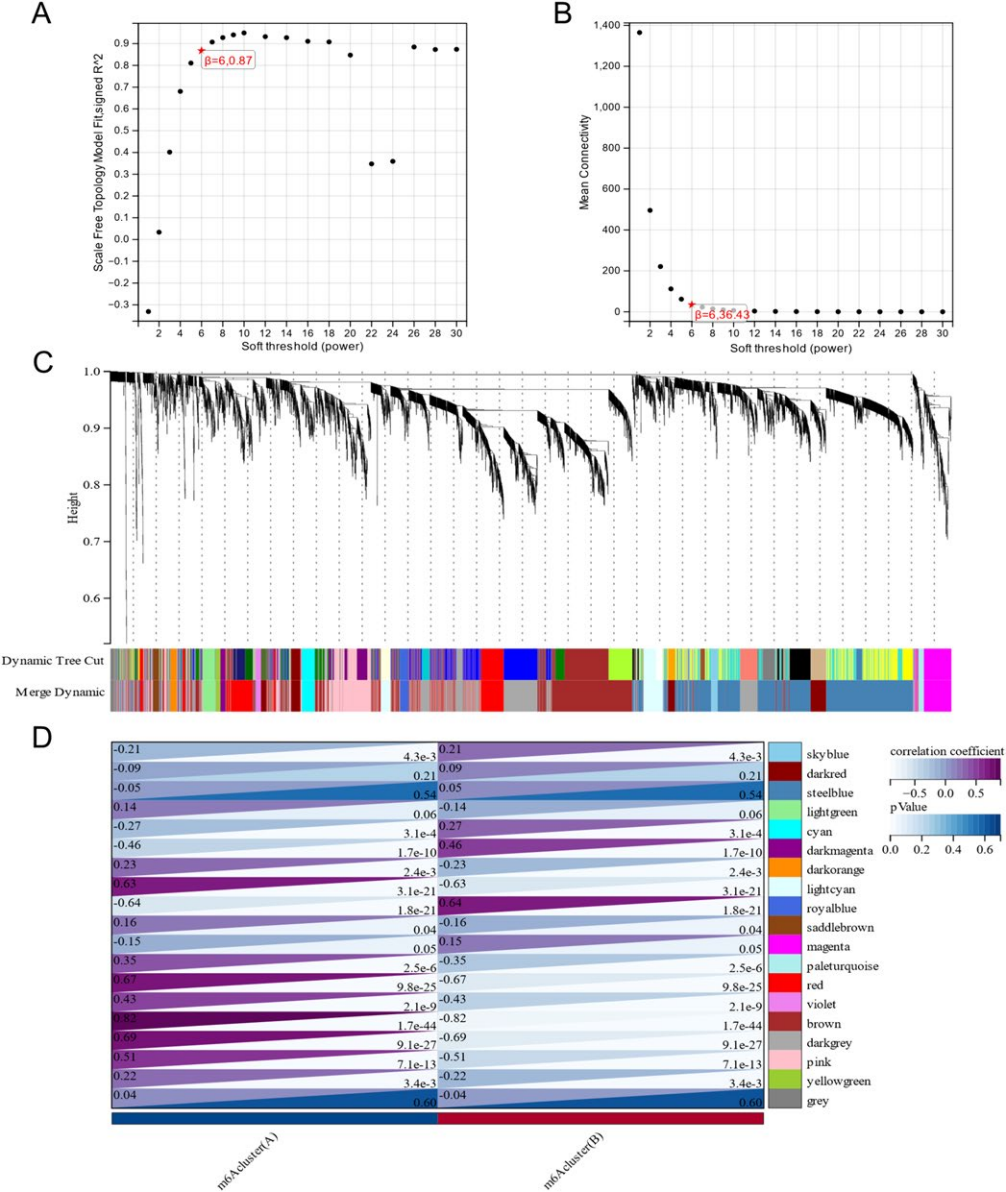
Figure 8 illustrates the Weighted Gene Coexpression Network Analysis (WGCNA) for m6A modification patterns in VTE: (**A**) Analysis of network topology for various soft-thresholding powers. **B** Mean connectivity analysis for soft-thresholding powers. **C** Gene dendrogram obtained by clustering dissimilarity based on topological overlap, with assigned module colors. **D** Module-trait relationships, correlating gene modules with m6A modification patterns

Using the previously mentioned degrees, we performed WGCNA. The scale-free coexpression network must reach a minimum soft threshold of 6 to be considered scale-free, as shown in Fig. 8A and B. As illustrated in Figs. 7C and 8C, we constructed a coexpression network using the ideal soft threshold and divided the genes into 19 network modules. The gray module is composed of genes that are not part of any other module. The brown module showed the highest correlation with the m6A modification pattern based on module-pattern correlation analysis (Fig. 8D).

**Fig. 9:**
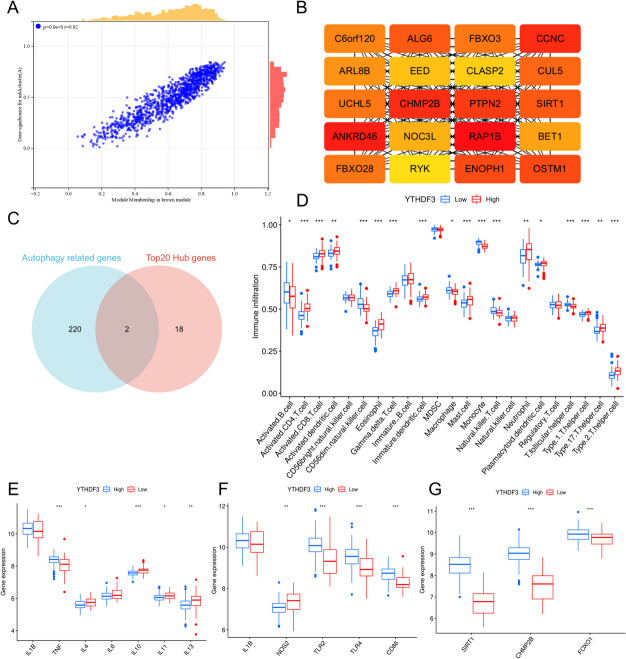
Figure 9 shows the analysis of the brown module’s correlation with m6A modification pattern and immune infiltration in VTE: (**A**) Scatterplot of module membership (MM) vs. gene significance (GS) in the brown module. **B** Protein-protein interaction network of the brown module’s hub genes. **C** Venn diagram of autophagy-related genes and top 20 hub genes. **D**-**G** Boxplots showing differences in immune infiltration and gene expression levels associated with high and low YTHDF3 expression

We further investigated the relationship between the brown module and the m6A modification pattern by determining the GS, MM, and gene expression of the module feature vector. Our findings demonstrated that MM and GS are positively correlated (Fig. 9A), indicating that the brown module also depends on the genes associated with the m6A modification pattern. Based on the similarity of the genes in the major modules, we used Cytoscape to build a protein–protein interaction (PPI) network and display the gene interactions (Fig. 9B) [46]. By comparing the significant genes in the brown module that met the condition (|MM| > 0.8, |GS| > 0.1) to the significant genes in the PPI network (node degree > 5), we identified reliable hub genes (Additional file 8). After analyzing the top 20 important genes and autophagy-related genes, we identified two hub genes, CHMP2B and SIRT1 (Fig. 9C). A recent study on mammalian cells has indicated that the endocytosis pathway, the successful fusion of autophagic vesicles, and the destruction of autophagic products depend on the endosomal sorting complex necessary for transport (ESCRT) activity [47]. Sirtuins are an evolutionarily conserved family of NAD+-dependent deacetylases and ADP-ribosyltransferases [48] that are involved in several biological functions [49]. SIRT1, one of the most sought-after sirtuins in mammals, affects numerous cellular and organismal functions, including metabolism, immunological response, and aging [50]. During senescence, the autophagy protein LC3 identifies SIRT1 in the nucleus as something to be destroyed. This occurs through autophagosome–lysosome interactions [51].

### Study on the mechanism of VTE caused by YTHDF3 by affecting the immune microenvironment and autophagy

Based on the aforementioned bioinformatics analysis, we merged the first five important genes (i.e., *YTHDF1*, *HNRNPC*, *ELAVL1*, *IGFBP1*, and *YTHDF3*) obtained from the nomogram prediction model and two genes (i.e., *YTHDF3* and *HNRNPA2B1*) that had the greatest correlation with the VTE immune microenvironment and obtained YTHDF3, which plays an important role in the VTE m6A modification mode and VTE immune microenvironment. Simultaneously, the sequencing results of the dataset showed high expression of YTHDF3 in patients with VTE. We separated the patients into two groups depending on their levels of YTHDF3 expression, with the median gene expression serving as the dividing line, to further examine the role of YTHDF3 in the occurrence and progression of VTE.

First, we compared the immune cell infiltration between the groups with high and low YTHDF3 expression. The high-expression group had a more prevalent mononuclear phagocyte system but lower neutrophil counts than the low-expression group (Fig. 9D). The clearance of pathogens and injured tissues and the activation and migration of neutrophils are crucial early phases of an inflammatory response [52]. Simultaneously, neutrophils release several inflammatory chemicals to initiate a cascade reaction [53]. In the low-expression group, the expression of TNF-β decreased, whereas the expression of the anti-inflammatory factors IL-4, IL-10, IL-11, and IL-13 increased dramatically (Fig. 9E). Low levels of YTHDF3 expression may limit the acute inflammatory response mediated by neutrophils. This reduction may lower the level of inflammatory reaction and the formation of arterial thrombosis, thereby lowering the risk of VTE and protecting cardiovascular health. The mononuclear phagocyte system is mainly involved in the middle and late phases of the inflammatory response [54]. Based on their roles and rates of releasing inflammatory factors, macrophages can be divided into two groups: M1 and M2 types [55]. The primary activators of M1 macrophages (classically activated macrophages) are LPS and IFN; these cells primarily increase inflammation, sterilization, and phagocytosis by releasing significant amounts of IL-2 and less-to-negligible amounts of IL-10. M2 macrophages (alternatively activated macrophages) release IL-10 and other anti-inflammatory cytokines, which aid in tissue regeneration and wound healing. This suppresses M1 macrophage activity. M2 macrophages are primarily stimulated by IL-4 [56]. According to an in vitro study conducted by Yi HaoWu et al., the selective deletion of YTHDF3 in macrophages can properly suppress macrophage M1 polarization and increase macrophage M2 polarization [57]. Comparison of the expression levels of macrophage-related genes in the high and low YTHDF3 expression groups revealed that the expression levels of proinflammatory M1 macrophage-related genes (i.e., *TLR2*, *TLR4*, and *CD86*) were decreased in the low expression group, except for NOS2 expression (Fig. 9F). This finding indicates that YTHDF3 generally promotes the inflammatory response mediated by macrophages, leading to the obstruction of venous thrombosis and reduction of the risk of thromboembolism caused by thrombus shedding.

In a previous study [58], KEGG analysis of DEGs revealed that autophagy played a major role in the differential gene function of two groups of patients with VTE under different m6A modification conditions. By analyzing the WGCNA network expression of DEGs, two HUB genes related to autophagy were identified: *SIRT1* and *CHMP2B*. By comparing the expression levels of autophagy-related genes between the two groups, it was observed that the expression levels of *SIRT1* and *CHMP2B* were relatively higher in the high YTHDF3 expression group than in the low YTHDF3 expression group. Studies [59] have shown that YTHDF3 mediates m6A modification by METTL3 to promote autophagy and that the absence of YTHDF3 damages autophagy formation and lysosomal function [60]. CHMP2B is the main component of the autophagy-related functional protein ESCRT-3, and the promotion of autophagy by YTHDF3 may correspondingly increase the expression of *CHMP2B*. Currently, *SIRT1* can regulate autophagy in two ways: [1] directly through deacetylation of the expression products of the autophagy-related genes *Atg5*, *Atg7*, and *Atg8* and [2] through activation of FoxO in the nucleus [61]. YTHDF3 may promote autophagy by inducing *SIRT1* overexpression, which should be examined in future studies. According to Francesco et al. [62], *SIRT1* overexpression increases the proliferation ability of bone marrow-derived macrophages during their differentiation. In contrast, the inactivation of shRNA, deletion mediated by CRISPR/Cas9, and drug inhibition have been reported to reduce the expression of *SIRT1*, thereby restricting the self-renewal of macrophages in culture. Simultaneously, *SIRT1* inhibits the negative regulation of the G1/S transition, cell cycle process, and self-renewal gene network [63]. This includes inhibition of E2F1 and Myc, activation of FoxO1 and SIRT1, and targeting SIRT1 to mediate the cell cycle process and stress response [62]. The analysis of the gene expression levels of the high and low YTHDF3 expression groups revealed that the FOXO1 level of the high YTHDF3 expression group was higher than that of the low YTHDF3 expression group (Fig. 9G). This proves that there may be a similar SIRT1–FOXO1 pathway in vivo that promotes macrophage proliferation, which may strengthen the inflammatory reaction and thus increase thrombosis. Inhibition of YTHDF3 function may reduce thrombosis by inhibiting the SIRT1–FOXO1 pathway, offering us a novel strategy for preventing VTE.

## Discussion

CVD is the most frequent cause of death worldwide [64]. Genetic and epigenetic variables play a crucial role in the development of CVD [65]. In recent years, as the mechanism of action of epigenetic factors, such as m6A RNA methylation modification, has gradually been clarified and research methods have gradually increased [66], an increasing number of researchers are investigating the effect of m6A methylation modification on CVD [67]. VTE, the third most common CVD, poses a significant threat to patients’ life expectancy and quality of life, making early prevention and diagnosis of the disease crucial. m6A methylation, which regulates the immunological milieu, may affect the physiological function of immune cells [68]. The formation of thrombosis is inseparable from the interaction between platelets and innate immune cells and the inflammatory response mediated by various cytokines [69–71]. We hypothesized that m6A alteration modulates the immunological microenvironment, thereby influencing the incidence and development of VTE.

To better stratify the risk of VTE, we developed an m6A rating system (VTE score) for DVT. We used PCA to quantify the differences in the methylation patterns of the 14 m6A methylation model characteristic genes related to the prognosis in every patient with VTE. Research on this scoring system shows that a higher score indicates a higher correlation between autophagy and the immune microenvironment. This indicates the precision and specificity of our methodology for evaluating VTE. ELAVL1 and REM15B were positively correlated with monocyte abundance, whereas YTHDF3, HNRNPC, and HNRNPA2B1 were negatively correlated with monocyte abundance. Macrophages were also correlated with six m6A regulatory factors (i.e., METTL3, RBM15, YTHDC2, YTHDF3, HNRNPA2B1, and IGFBP3) (Fig. 4C). Inflammation was positively correlated with HNRNPA2B1, HNRNPA2C3, and ZNFHB3. Neutrophils are the core immune cells in acute inflammation [72, 73]. They were negatively correlated with LRPPRC and ELAVL1. Based on the results of these analyses, we identified two genes (i.e., YTHDF3 and HNRNPA2B1) among the m6A methylation modification regulators that are most closely associated with inflammatory responses. HNRNPA2B1, a heterogeneous nuclear ribonucleoprotein [74], is a member of the A/B subfamily. Heterogeneous nuclear RNA is bound by hnRNPs, which are RNA-binding proteins (hnRNA) [75]. Pre-mRNA processing and other facets of mRNA metabolism and transport are thought to be regulated by the proteins involved in this process, which are connected to pre-mRNAs in the nucleus [76]. Researchers have discovered that hnRNPA2B1 regulates innate immunity [77]. SIRT1 modulates inflammation via its NAD-dependent deacetylase and posttranslational regulation [78]. Through the deacetylation of transcription factors and histones, SIRT1 controls inflammation, apoptosis/autophagy, aging (life span and health span), calorie restriction/energetics, mitochondrial biogenesis, stress resistance, cellular senescence, endothelial functions, and circadian rhythm [79].

The regulatory impact of the m6A modification modes of linked genes on the VTE immune microenvironment was clarified using an unsupervised clustering method based on m6A regulators. Based on 14 m6A modulators, we could distinguish between two m6A methylation modification modes. These modes displayed varying levels of m6A regulatory factor expression and immune microenvironment features. These findings suggest future lines of inquiry for biological experimentation. Mode B had a higher concentration of infiltrated-activated B cells and CD56 + natural killer cells and slightly higher levels of mononuclear macrophages than mode A. In contrast, mode A had higher levels of activated CD4 + T cells, activated CD8 + cells, eosinophils, immature B cells, neutrophils, dendritic cells, and various T helper cells. By analyzing data from the HGNC database [34], we observed a difference in the expression of genes related to the inflammatory response between the two m6A modification modes. The mode B immunophenotype was associated with an increased intravascular inflammatory response, which may lead to more vascular thrombosis and a worse prognosis. Gene function analysis revealed that these m6A-related genes were associated with the autophagy signaling pathway. Under physiological conditions, autophagy is a critical process for intracellular homeostasis maintenance and cell remodeling [80]. Moreover, autophagy is crucial for preserving the intracellular homeostasis of cardiomyocytes, endothelial cells, and arterial smooth muscle cells and is closely related to the onset of CVD [81]. Through WGCNA and topological analysis, we finally selected two pivotal genes, *CHMP2B* and *SIRT1*. However, MeRIP-seq and cytological tests are still required to confirm these projected key genes. A component of ESCRT-3 is encoded by *CHMP2B* [82]. Both the efficient fusion of autophagy vesicles with endocytotic pathways and the destruction of autophagy products depend on functional ESCRTs [47]. NAD+-dependent deacetylases and ADP-ribosyltransferases of the Sirtuin family [83], which have shown evolutionary conservation, include SIRT1 [84]. SIRT1 is essential for several cellular and organismal functions, including aging, immunological response, and metabolism [50]. SIRT1 is an autophagy substrate that is broken down by autophagosome–lysosome interactions during senescence and is recognized by the autophagy protein LC3 [51].

Based on the aforementioned bioinformatics analysis, we identified the m6A methylation regulatory modifier gene YTHDF3, which has the strongest correlation with the immune microenvironment and autophagy. We explored two pathways through which YTHDF3 may influence the occurrence and progression of VTE. The first pathway was related to the immune microenvironment of VTE. Through different methylation modification modes of m6A, the abundance of neutrophils was higher in the group with high YTHDF3 expression. Furthermore, macrophages were more differentiated into the proinflammatory M1 type, which enhanced the inflammatory response and further increased the possibility of VTE. The second possible mechanism through which YTHDF3 affects VTE is autophagy enhancement, which indirectly affects thrombus formation. In the starvation state, YTHDF3 promotes autophagy in mice through methylation modification of m6A, whereas two HUB genes related to autophagy (i.e., CHMP2B and SIRT1) have a significant increase in expression with the promotion of autophagy by YTHDF3 [82]. At present, SIRT1 has been demonstrated to modulate autophagy through two mechanisms: direct deacetylation of the autophagy-related genes Atg5, Atg7, and Atg8 and activation of FoxO in the nucleus [51]. YTHDF3 may regulate the SIRT1 autophagic pathway, which should be verified by subsequent experiments. Recently, in vitro experiments have demonstrated that the overexpression of SIRT1 enhances the proliferation of bone marrow-derived macrophages during differentiation [62]. By analyzing the gene expression levels of the high and low YTHDF3 expression groups, we concluded that the level of FOXO1 in the high YTHDF3 expression group was higher. This suggests a similar SIRT1–FOXO1 pathway in vivo that promotes the proliferation of macrophages, thus strengthening the inflammatory response and increasing thrombosis.

To the best of our knowledge, no studies to date have examined the function of m6A alterations in VTE. The findings of the present study revealed that autophagy and the immune microenvironment may be related to m6A alterations in VTE. We obtained encouraging findings by merging data from public databases with our sequencing data. The findings of this study may contribute to the elucidation of the complicated pathophysiological pathways underlying excessive proinflammatory immune cell activation and an elevated inflammatory response in VTE. Moreover, these findings provide new avenues for research into the prevention, diagnosis, risk assessment, and treatment of VTE.

Based on the m6A-related biomarkers identified in this study, we can develop clinical models for predicting the risk of VTE occurrence. Through prospective cohort studies, we can collect peripheral blood samples from VTE patients and healthy controls, detect the expression profiles of m6A regulators and related genes, and combine this information with demographic characteristics, clinical risk factors, lifestyle, and other data to establish a multifactorial VTE risk prediction model. This model can incorporate machine learning algorithms, optimize model parameters through training sets, and evaluate its predictive performance on validation and test sets to obtain a reliable risk assessment tool. This tool can be applied in clinical settings for risk screening in healthy populations or suspected VTE patients, identifying high-risk individuals, which can help in timely intervention and reduce the occurrence of VTE. For example, in high-risk populations such as those with cancer or undergoing orthopedic surgery, routine testing of m6A biomarkers can be performed, and combined with clinical models to assess their risk of developing VTE. Preventive anticoagulation therapy can then be administered to high-risk individuals, thereby reducing the incidence and mortality of VTE.

The m6A regulators and related genes identified in this study hold promise as novel blood-based biomarkers for prognostic assessment in VTE patients. We found that the expression profile of m6A writers, erasers, and readers in the peripheral blood of VTE patients exhibits unique alteration patterns and is closely associated with the level of immune cell infiltration. This suggests that by detecting the expression profile of m6A regulators in the peripheral blood of VTE patients, we can assess the m6A modification level and inflammatory status in patients, thereby determining disease severity and prognosis. For example, based on our results, high expression of YTHDF3 may indicate increased neutrophil and inflammatory monocyte infiltration, stronger inflammatory response, and potentially worse prognosis. The combination of these biomarkers could be incorporated into routine blood tests for VTE patients, assisting in disease stratification and personalized treatment planning. Furthermore, dynamically monitoring changes in m6A biomarkers in patients’ blood can help evaluate disease progression and the efficacy of therapeutic interventions, guiding clinical decision-making. We can also integrate the m6A expression profile with other clinical indicators, such as D-dimer and C-reactive protein, to establish multi-omics prognostic prediction models, further enhancing the precision of VTE prognostic assessment. Of course, these ideas need to be validated in large-sample prospective cohorts, and standardized detection processes should be developed to promote the clinical translation of m6A biomarkers.

This study also provides new insights into precise stratification and personalized treatment for VTE patients. m6A epigenetic modification is involved in regulating multiple disease-related pathways and may affect patients’ responsiveness to conventional treatments such as anticoagulation and thrombolysis. By detecting m6A regulators and target genes, we may identify patient subgroups with different m6A modification patterns and predict their treatment responsiveness, enabling molecular subtyping-guided personalized precision therapy. For example, in patients with high YTHDF3 expression and hyperinflammation, the combination of anti-inflammatory drugs could be attempted; while in patients with abnormal expression of autophagy genes such as SIRT1, the combination of autophagy modulators could be considered. Moreover, m6A regulators themselves may also become new therapeutic targets for VTE. Our study suggests that inhibiting YTHDF3 can attenuate inflammatory responses, and specific YTHDF3 inhibitors may be developed for VTE precision treatment in the future. Epigenetic therapies targeting m6A abnormalities may bring new benefits to patients with refractory or recurrent VTE, but their long-term efficacy and safety require further investigation. In summary, m6A modification pattern analysis may guide the optimization and combination of personalized treatment plans for VTE, offering patients more precise and effective treatment options.

In summary, this study has broad application prospects in the clinical diagnosis and treatment of VTE. Firstly, we can develop m6A-based prediction models to assess patients’ risk of developing VTE. Secondly, by detecting the expression profile of m6A regulators in peripheral blood, we can evaluate the prognosis of VTE patients and guide treatment decisions, thereby achieving molecular subtype-guided personalized precision therapy. This will help improve the long-term prognosis of VTE patients and reduce the disease burden. In the future, we can further integrate multi-omics data such as genomics, transcriptomics, and proteomics to construct VTE precision medicine models and clinical decision support systems, integrating multi-dimensional information including m6A epigenetic modification, immune microenvironment, and autophagy status, comprehensively enhancing the level of VTE diagnosis and treatment. We look forward to more studies validating our findings and accelerating the translational application of m6A research achievements in VTE and other cardiovascular diseases, benefiting a wider range of patient populations.

### Supplementary Information

**Additional file 1.** Data banks/repositories corresponding to all datasets analyzed in this study.

**Additional file 2.** The list of all m6A regulators.

**Additional file 3.** The list of gene sets of 23 immunocytes.

**Additional file 4.** The list of gene sets of immune responses.

**Additional file 5.** The list of major histocompatibility complex (MHC)-related genes.

**Additional file 6.** Venn diagram.

**Additional file 7.** Sample grouping information.

**Additional file 8.** Hub Genes.

## Data Availability

The analyses conducted in this study utilized R version 4.2.2 (https://cran.rstudio.com/), a widely used open-source programming language and software environment for statistical computing and graphics.
